# A prospective clinical study of the implications of IL-8 in the diagnosis, aggressiveness and prognosis of prostate cancer

**DOI:** 10.4155/fsoa-2017-0084

**Published:** 2017-11-15

**Authors:** Thierry Roumeguère, Francois Legrand, Elie El Rassy, Mehdi Idrissi Kaitouni, Simone Albisinni, Alexandre Rousseau, Michel Vanhaeverbeek, Sandrine Rorive, Christine Decaestecker, Olivier Debeir, Karim Zouaoui Boudjeltia, Fouad Aoun

**Affiliations:** 1 Department of Urology, University Clinics of Brussels, Hôpital Erasme, Université Libre de Bruxelles (ULB), Brussels, Belgium; 2 Laboratory of Experimental Medicine, Unit 222 ULB, Hôpital André Vésale, CHU Charleroi, Montigny-le-Tilleuil, Belgium; 3 Department of Oncology, Hôtel Dieu de France University Hospital, Faculty of Medicine, Saint Joseph University, Beirut, Lebanon; 4 Department of Pathology, University Clinics of Brussels, Hôpital Erasme, ULB, Brussels, Belgium; 5 DIAPath – Center for Microscopy & Molecular Imaging (CMMI), ULB, Gosselies, Belgium; 6 Multimodal Image Processing units – Center for Microscopy & Molecular Imaging (CMMI), ULB, Gosselies, Belgium; 7 Laboratory of Image, Signal processing & Acoustics, Brussels School of Engineering/École Polytechnique de Bruxelles, ULB, Brussels, Belgium; 8 Department of Urology, Hôtel Dieu de France University Hospital, Faculty of Medicine, Saint Joseph University, Beirut, Lebanon

**Keywords:** aggressiveness, biochemical recurrence, diagnosis, IL-8, prostate biopsy, prostate cancer, radical prostatectomy

## Abstract

**Aim:**

We evaluated the relationship between IL-8 and prostate cancer (PCa) with emphasis on diagnosis, aggressiveness and prognosis.

**Materials & methods:**

Prostate-specific antigen (PSA) and serum IL-8 were collected from patients undergoing prostate biopsy. IL-8 expression was evaluated on immunohistochemistry with IL-8 labeling index. Complete follow-up of this cohort was achieved over a period of up to 6 years with continuous follow-up of PSA levels.

**Results:**

Among 135 patients, serum IL-8 level did not correlate to the diagnosis or aggressiveness of PCa. In 52 radical prostatectomy specimens, a higher IL-8 labeling index was detected in the tumor areas (0.4 ± 0.2 vs 0.33 ± 0.2; p = 0,007) but did not correlate to any of the prognostic markers: D'Amico classification (p = 0.52), Gleason score (p = 0.45), perineural (p = 0.83) and capsular invasion (p = 0.75). No correlation was found to PSA biochemical-free failure.

**Conclusion:**

IL-8 serum level was not a significant predictor of diagnosis, aggressiveness or prognosis of PCa.

During the last 15 years, prostate cancer (PCa) has become the leading cause for cancer incidence in men with 1.4 million new cases per year [1]. These stems mainly from the widespread use of serum prostate-specific antigen (PSA)-based PCa screening. However, the limited specificity of PSA and the poor sampling of cancers under 2D transrectal ultrasonography guided biopsy have led to subsets of patients with significant disease being undiagnosed and others with clinically insignificant disease were unnecessary treated [2]. Therefore, mortality from PCa has remained consistent [3]. At present, the great challenge is the development of more accurate diagnostic strategies to better risk-stratify patients and to facilitate clinical decision making. This is a timely issue given the recent data on genetic and biologic behavior of prostatic cancer foci [4]. It is also frequently postulated that chronic prostatitis is a risk factor for the development of PCa implying that the selective inhibition of inflammatory cascade would thus prevent PCa development and progression [5]. However, there is no definitive proof for this assumption.

Higher IL-8 expression has been reported in cancerous prostatic culture cells compared with hyperplastic and normal epithelium [6]. IL-8 is a proinflammatory chemokine produced by macrophages and epithelial cells which promotes angiogenesis. Subsequently, higher serum levels of IL-8 have been shown in patients with PCa compared with healthy volunteers. Serum IL-8 levels were found to be correlated with increasing stages of PCa and were useful to discriminate cancer from benign prostatic hyperplasia, suggesting that IL-8 serum assay could have a clinical value in daily practice [7]. A stepwise rising in IL-8 expression with advanced pathological stage and high Gleason score was also documented [8]. Recently, significantly higher levels of IL-8 expression in patients with aggressive PCa or presenting with biochemical recurrence have been reported [9]. IL-8 has also been considered as a molecular determinant of androgen-independent PCa [10]. However, it remains undetermined whether IL-8 could play a role in prostate carcinogenesis and disease progression.

The aim of the present study was to analyze the relationship between serum IL-8 measurements and PCa with emphasis on diagnosis, aggressiveness and prognosis from prebiopsy conditions to PSA biochemical-free failure.

## Materials & methods

This prospective study was conducted at Hôpital Erasme-ULB, Belgium between 2008 and 2011. We included all treatment-naive patients scheduled for their first prostate biopsy. Patients with previous or current radiation or hormonal treatment could not be included in this study. Patients with autoimmune and chronic inflammatory diseases or active inflammation (sever chronic bronchitis, hepatitis, high-grade esophagitis and pancreatitis) or neoplasms (melanoma and tonsillar squamous cells carcinoma) were excluded. All patients signed an informed consent before enrollment and the study was approved by the Ethical Committee of the Brussels University Clinics, Hôpital Erasme-ULB, Brussels, Belgium.

Peripheral blood was collected prior to the prostate biopsy. Sera were obtained after centrifugation and stored at -25°C. PSA measurements were quantitated by the commercially available system (BD Biosciences, NJ, USA). IL-8 measurements were made on each serum sample using highly sensitive commercially available enzyme-linked immunosorbent assay (BD Opt EIA Human IL-8 ELISA Kit II, BD Biosciences) according to the manufacturer's instructions and performed by the Laboratory of Experimental Medicine, 222 unit ULB at the CHU de Charleroi (site Vésale, Belgium). Patients underwent 2D transrectal ultrasound-guided sextant biopsy and two additional transition zone biopsies, after local injection of 10 ml lidocaine 1% and antibioprophylaxis. Prostate volume was measured to compute PSA density (PSAd), the ratio between PSA and prostate volume. All the samples (prostate biopsies and radical prostatectomy [RP] specimen) were reviewed by the same pathologist for cancer diagnosis, Gleason score assessment (International Society of Pathology [ISUP], 2005), pathological stage according to the International Union for Cancer Control (IUCC, 2009) classification, surgical margin status and perineural and lympho-vascular involvement status.

Four human tissue microarrays (TMAs) were manufactured using archival formalin-fixed and paraffin-embedded samples from RP specimens. For each patient, sample cores (diameter of 600 μm) were taken from both tumor areas and normal parenchyma distant from the tumor zone. Standard immunohistochemistry (IHC) was applied to single 5 μm-thick sections from TMA using an IL-8 antibody to display IL-8 expression. IL-8 monoclonal antibody samples utilized in the study was a mouse monoclonal IgG2b K antibody raised against a recombinant protein corresponding to epitope 40–99 mapping at the carboxy terminus of IL-8 of human origin (Santa Cruz Biotechnology, TX, USA). After slide digitization (using NanoZoomer HT 2.0, Hamamatsu, Hamamatsu city, Japan), the correct assignment of the core images in the TMA grid was done using an *ad hoc* algorithm, as previously described [11]. The same uropathologist validated each core individually for sample quality (e.g., no folding or detachment), diagnosis (i.e., tumoral and peritumoral) and for removing artifacts (e.g., areas with marked macrophages). For each patient, we computed the IL-8 labeling index (IL-8 LI), which is the percentage of immunostained tissue area compared with total tissue area, in tumor and peritumor areas separately. To obtain the tissue area, we used the optical densities (OD) in the red, green and blue channels of the slide image using the standard OD = log (I/I_0_) definition, where I_0_ is the mode of the pixel intensities in the glass slide area corresponding to the white background. A pixel is considered as belonging to the tissue if at least one of the three OD values is higher than a small value set experimentally to 0.1. The DAB (3,3′ diaminobenzidine tetrahydrochloride; a product used in HRP-basedassays suitable for immunohistochemical staining) channel is then extracted from the OD-converted red, green and blue color image by means of color deconvolution [12]. The IHC-stained surface is defined as pixels where the DAB OD is higher than 0.1 (the threshold was set experimentally, validated by the pathologist and kept constant during the complete experiment).

Biochemical recurrence was defined as a PSA level of >0.2 ng/ml on two successive measurements after RP. The first date of elevated PSA was considered the date of recurrence. Complete follow-up of this cohort was achieved over a period of up to 6 years.

### Statistical analysis

All analyses and graphics were performed using the XL Stata software (Addinsoft version 2010). Kolmogorov–Smirnov test was applied and rejected normality for IL-8 serum assay, IL-8 LI and PSA data. Mann–Whitney and Spearman's correlation tests were used for comparison between samples with nonparametric skewed variables. The receiver operating characteristic (ROC) curves were generated to look for a predictive power. The X^2^ value generated by the logistic regression models is also presented to affirm the statistical power of each model. Probabilities for the time to biochemical failure were calculated using standard Kaplan–Meier analysis and the multivariate Cox proportional hazards regression. All results are presented as mean ± SD or median and the interquartile range (Q25–Q75). A two-sided p < 0.05 defined statistical significance.

## Results

**Figure 1.**
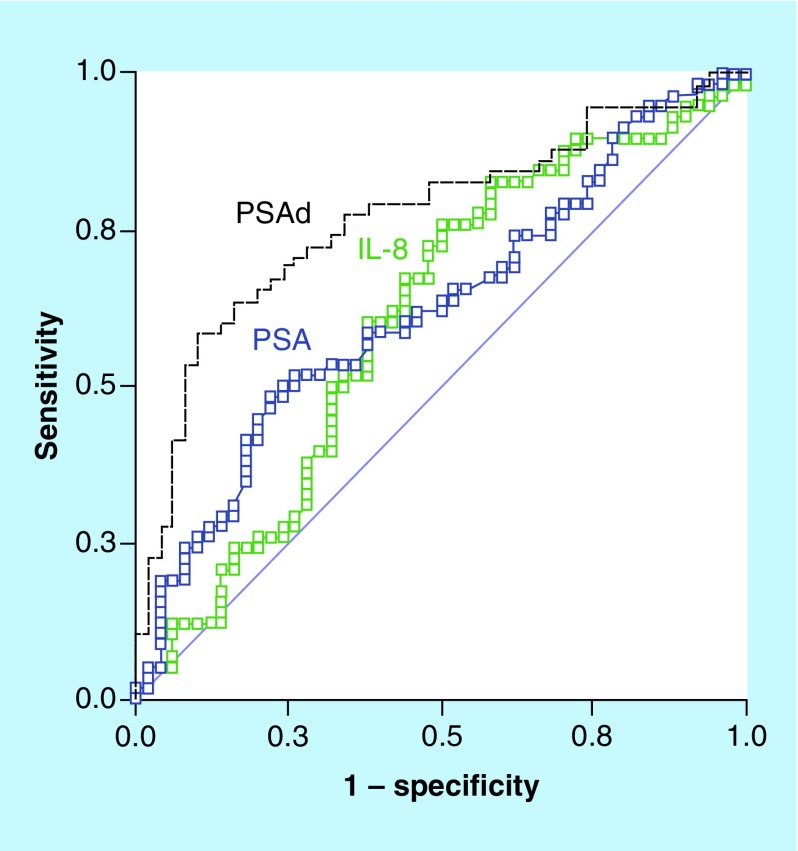
**Receiver operating characteristic curves modeling the probability of distinguishing prostate cancer from healthy subjects.** Measure equivalent to the area under the curve (AUC). Strongest predictors of having prostate cancer are PSA with AUC = 0.61 (p < 0.05) and PSA density with AUC = 0.76 (p < 0.05). IL-8 serum levels is not predictive of prostate cancer with AUC = 0.61 (p = 0.12). PSA: Prostate-specific antigen.

Overall, our study included 145 patients with 10 being excluded for meeting exclusion criteria. The characteristics of the study sample are illustrated in Tables 1 & 2. Among the 135 patients, who underwent prostate biopsies, 77 were diagnosed of PCa (57%). No significant difference was detected in serum IL-8 measurements between patients with or without histologically proven PCa (p = 0.13). No correlation was found between IL-8 and age to discriminate PCa patients as patient's ages were not different between patients with positive or negative biopsies (p = 0.03). Figure 1 provides an array of ROC curves and the AUC for the ability of PSA, PSAd and IL-8 serum assay to detect PCa. Unlike PSA and PSAd, IL-8 was unable to detect patients with histology-proven PCa (AUC = 0.61 [p < 0.05], 0.76 [p < 0.05], 0.61 [p = 0.12], respectively). Furthermore, no ability was found in discriminating patients with more aggressive disease (Gleason score ≥7 on biopsy or PSA >10 ng/ml) from others. None of patients with initial negative biopsy has been diagnosed of PCa or any other malignancy during the study period.

**Table 1.** **Preoperative characteristics of population samples.**

**Characteristics**	**Biopsy**	**Biopsy -**	**Biopsy +**	**Pathology by radical prostatectomy**
n	135	68	77	52

Age (years)^†^	63 ± 8	61 ± 7	65 ± 8	63 ± 7

BMI (kg/m^2^)^†^	27 ± 4	27 ± 3	27 ± 4	41 ± 15

Total prostate volume (ml)^†^	51 ± 26	61 ± 29	41 ± 15^§^	41 ± 15

Transition zone volume (ml)^†^	26 ± 17	32 ± 21	21 ± 12	21 ± 13

PSA (ng/ml)^‡^	6.8 (5.1–10.0)	6.1 (4.3–7.9)	7.9 (5.5–12.3)^§^	7.7 (5.5–11.2)

PSA density (ng/ml^2^)^‡^	0.15 (0.10–0.23)	0.11 (0.09–0.15)	0.20 (0.13–0.30)^§^	0.19 (0.13–0.29)

Serum IL-8 (pg/ml)^‡^	5.4 (3.8–8.2)	4.8 (2.8–8.0)	6.4 (4.5–8.6)	6.6 (4.7–8.4)

^†^Mean ± SD.

^‡^Median (Q25–Q75).

^§^Significantly different from biopsy-negative proven prostate cancer.

Biopsy: Patient undergoing prostate biopsy; Biopsy -: Patient without biopsy-proven prostate cancer; Biopsy +: Patient with biopsy-proven cancer; n: Number of patient; PSA: Prostate-specific antigen; RP: Patient undergoing radical prostatectomy.

**Table 2.** **Postoperative characteristics of the patients that underwent radical prostatectomy.**

**Characteristics**	**Pathology**	**Number of patients (%)**
Gleason score	6	18 (34%)

	7 (3 + 4)	19 (37%)

	7 (4 + 3)	9 (17%)

	8	2 (4%)

	9	4 (8%)

Nodes status	Negative nodes	50 (96%)

	Positive nodes	2 (4%)

Tumor stage	≤pT2b	5 (9%)

	pT2c	28 (54%)

	pT3a	15 (29%)

	≥pT3b	4 (8%)

D'Amico risk category	Intermediate risk	5 (10%)

	High risk	47 (90%)

PSA biochemical recurrence	Yes	17 (33%)

	No	35 (67%)

PSA: Prostate-specific antigen.

After stratifying the 52 patients that underwent RP according to the D'Amico classification, IL-8 levels were not statistically different between the D'Amico risk groups. In these subgroups, IL-8 did not correlate to the Gleason score (p = 0.26) or to the intraprostatic perineural invasion (p = 0.83) but correlated to capsular invasion (p = 0.02). With a single investigator selected cut-off value of 6.37 pg/ml, patients with higher IL-8 serum levels were more likely to present a capsular invasion with an odds ratio of 8.8 (95% CI: 2.5–30.5; p = 0.001), a negative predictive value of 81% and a positive predictive value of 67%.

**Figure 2.**
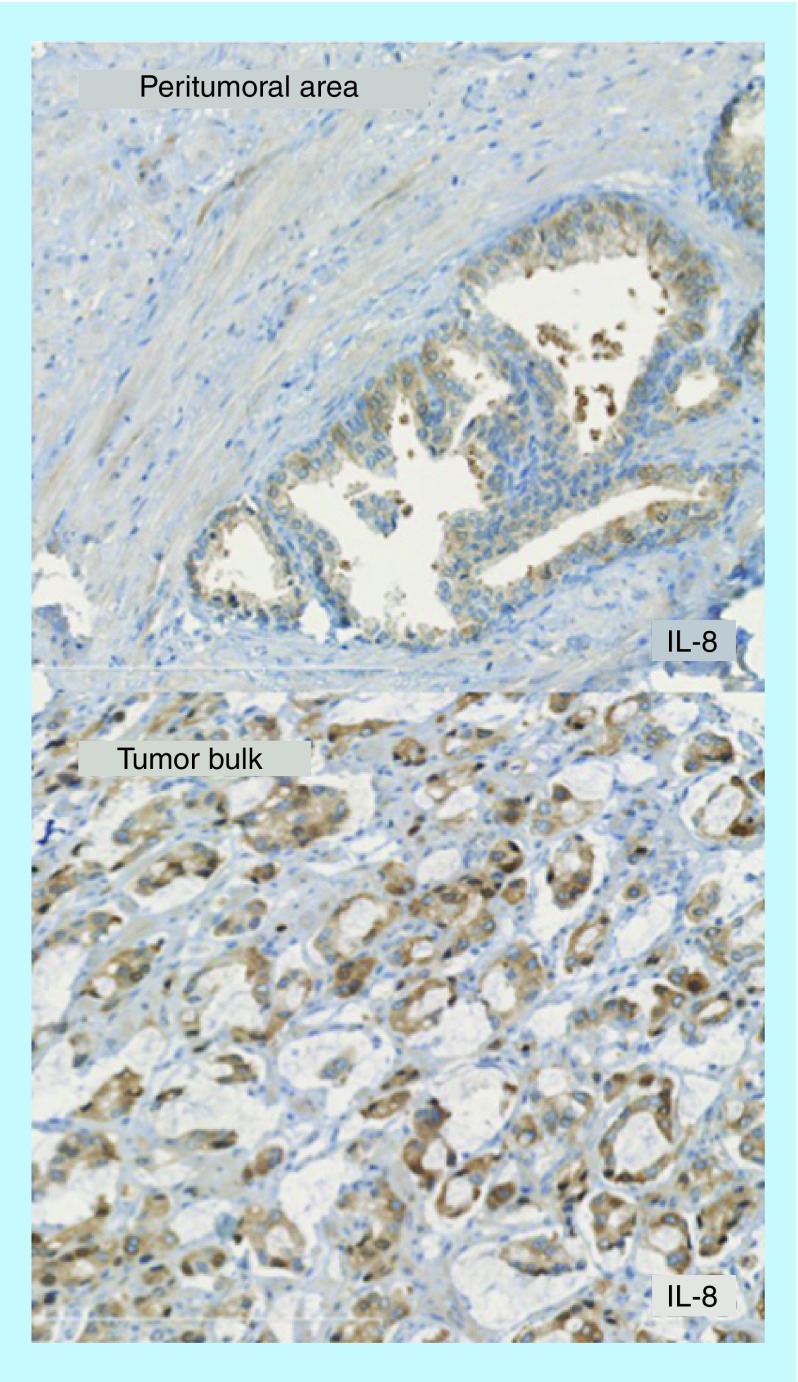
**Immunohistochemical expression of IL-8 in the peritumoral area and the tumor bulk from the same radical prostatectomy specimen.** The computed IL-8 label index showed higher expression in the tumor bulk compared with peritumoral area (p < 0.001).

IHC staining for IL-8 is illustrated in Figure 2. In the 52 RP specimens, a higher IL-8 LI was detected in the tumor zones than in noncancerous areas (0.4 ± 0.2 vs 0.33 ± 0.2; p = 0.007). IL-8 LI did not correlate to IL-8 serum levels (Spearman's correlation test, rho = 0.08; p = 0.03) or to any of the prognostic factors including the D'Amico classification (p = 0.52), the Gleason score (p = 0.45), the perineural (p = 0.83) and capsular invasion (p = 0.75).

**Figure 3.**
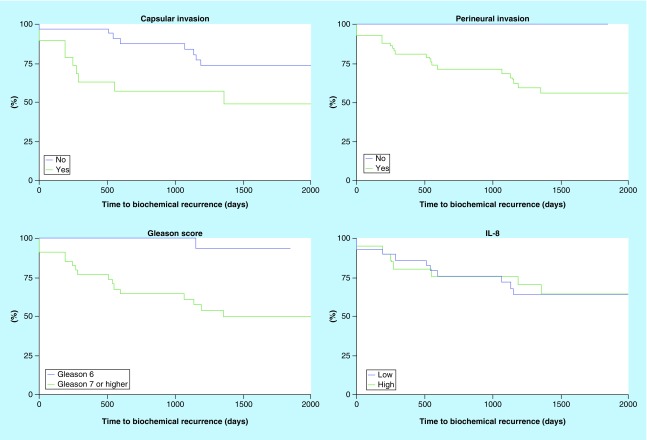
**Kaplan–Meier actuarial analysis for time to prostate-specific antigen biochemical recurrence.** The capsular and perineural invasion and the Gleason score were correlated to biochemical recurrence (Log-Rank p < 0.05). We defined low and high level of IL-8 according to the threshold 6.731 pg/ml defined by the ROC curve. Biochemical recurrence did not correlate to IL-8 (Log-Rank p > 0.05). ROC: Receiver operating characteristic.

Among the patients who underwent RP, 17 patients (33%) presented with PSA biochemical-free failure after a median duration of 43 months. The PSA biochemical-free failure correlated to perineural invasion, capsular invasion and Gleason score ≥7 in the univariate standard Kaplan–Meier analysis (Log-Rank p < 0.05) but significance was lost throughout the multivariate Cox proportional hazards analysis (p > 0.05). In both models, IL-8 serum levels and IL-8 LI did not correlate to PSA biochemical-free failure (Figure 3).

## Discussion

Many studies have demonstrated a role for IL-8 in the angiogenesis, tumoregenicity and metastasis of tumors [13–15]. More recently, the expression of IL-8 signaling has been shown to regulate the transcriptional activity of the androgen receptor in PCa and leading to castration resistant PCa [16]. This paper focuses on analyzing the role of IL-8 in the diagnosis and management of patients with early stage PCa.

### IL-8 & PCa diagnosis

The role of IL-8 in the diagnosis of PCa has never been confirmed. Initially, *in vitro* studies showed an increased IL-8 expression in cancerous prostatic cells in comparison to normal epithelium [6]. Later *in vivo* studies confirmed that IL-8 serum levels were higher among PCa patients and correlated to the staging at diagnosis [7]. More recently, a study designed to identify biomarkers for PCa failed to confirm a role for IL-8 in diagnosing PCa [17]. Our findings are consistent with these last results showing no contribution for IL-8 serum assay to discriminate PCa from benign disease in a subset of patients eligible for prostate biopsy. The discrepancy between results may be mainly attributed to the higher percentage of locally advanced and metastatic PCa (27% of PCa patients), known to present with higher inflammatory cytokines levels, found in Veltri *et al*. study [7]. In the same idea, this may considerably limit the potential impact of IL-8 measurement on a decision-making process in daily practice for patients presenting with autoimmune and chronic inflammatory diseases (excluded from our analysis).

### IL-8 & PCa aggressiveness

Experimental data reported significantly higher levels of IL-8 expression in primary cultured cells in patients with aggressive PCa (pathological stage ≥pT2c or Gleason score ≥7) in comparison to patients with nonaggressive PCa (pathological stage <pT2c, Gleason score 6) [9]. These findings were confirmatory of earlier data where a Gleason score ≥7 and a tumor score ≥pT3 correlated to higher IL-8 mRNA expression [8]. In our series, IL-8 serum levels and IL-8 IHC staining correlated to capsular invasion only suggesting a relationship between IL-8 and tumor stage. One explanation is that most of the studies, which reported a positive correlation between IL-8 and PCa, were based on advanced pathological disease contrary to our patients characteristics at earlier stage.

### IL-8 & biochemical recurrence after RP

The staining levels of PCa and microenvironment for IL-8 IHC have been reported higher in patients who developed later biochemical recurrence [9,18]. Finally, higher levels of IL-8 were also reported in patients who recurred [19]. In the present study, biochemical recurrence did not correlate to IL-8 serum levels or IL-8 LI and was limited to the traditional prognostic factors including perineural invasion, capsular invasion and Gleason score ≥7 on pathological examination. Neither IL-8 staining levels nor IL-8 serum assay could help in discriminating local or systemic recurrence.

This study included patients eligible for prostate biopsy, introducing *per se* a selection bias and results cannot be generalized to other situations. These results partially differ from previous published results and direct comparison remains uneasy because of studies with various methodologies and design, with heterogeneous populations including healthy volunteers or patients with more advanced disease. Due to a low specificity, increases in IL-8 levels may occur because of underlying inflammatory disease and attention must be paid to a relative instability of the IL-8 serum measurement especially in samples with relatively low concentrations of IL-8.

## Conclusion & limitations

In conclusion, our results did not describe a utility of IL-8 in early stage PCa. IL-8 serum level was not a significant predictor of diagnosis, aggressiveness or prognosis of PCa. The inconsistency between the IL-8 serum level and IL-8 LI may be explanatory of the lack of positive correlations. Furthermore, the majority of clinical studies reported the overexpression of IL-8 in the advanced stages that were limited in our sample.

## Future perspective

Increased expression of IL-8 has been characterized in cancer cells and tumor-associated macrophages, suggesting that IL-8 may function as a significant regulatory factor within the tumor microenvironment. As a consequence of the diversity of downstream targets, IL-8 signaling could promote angiogenesis, proliferation and survival of cancer cells, and potentiate the migration of cancer cells as well as infiltrating neutrophils at the tumor site.

However, the induction of IL-8 signaling is not well defined and due to the lack of specificity of IL-8, more research to establish the link between inflammation and early prostate carcinogenesis is mandatory.

**Summary points**

- Although reported mostly in advanced prostate cancer (PCa), we were not able to show an over expression of IL-8 serum level helping in the diagnosis of PCa at early stage.
- Further research is mandatory to understand the exact link between pro-inflammatory cytokines and prostate cancer.

## References

[1] Fitzmaurice C, Dicker D, Pain A (2015). The global burden of cancer 2013. *JAMA Oncol.*.

[2] Roehl KA, Antenor JA, Catalona WJ (2002). Serial biopsy results in prostate cancer screening study. *J. Urol.*.

[3] Siegel RL, Miller KD, Jemal A (2016). Cancer statistics, 2016. *CA Cancer J. Clin.*.

[4] Liu W, Laitinen S, Khan S (2009). Copy number analysis indicates monoclonal origin of lethal metastatic prostate cancer. *Nat. Med.*.

[5] Hung SC, Lai SW, Tsai PY (2013). Synergistic interaction of benign prostatic hyperplasia and prostatitis on prostate cancer risk. *Br. J. Cancer*.

[6] Ferrer FA, Miller LJ, Andrawis RI (1998). Angiogenesis and prostate cancer: *in vivo* and *in vitro* expression of angiogenesis factors by prostate cancer cells. *Urology*.

[7] Veltri RW, Miller MC, Zhao G (1999). Interleukin-8 serum levels in patients with benign prostatic hyperplasia and prostate cancer. *Urology*.

[8] Uehara H, Troncoso P, Johnston D (2005). Expression of interleukin-8 gene in radical prostatectomy specimens is associated with advanced pathologic stage. *Prostate*.

[9] Neveu B, Moreel X, Deschênes-Rompré MP (2014). IL-8 secretion in primary cultures of prostate cells is associated with prostate cancer aggressiveness. *Res. Rep. Urol.*.

[10] Araki S, Omori Y, Lyn D (2007). Interleukin-8 is a molecular determinant of androgen independence and progression in prostate cancer. *Cancer Res.*.

[11] Van Eycke YR, Debeir O, Verset L, Demetter P, Salmon I, Decaestecker C (25–29 August 2015). High-throughput analysis of tissue-based biomarkers in digital pathology. *Proceedings of The 37th Annual International Conference of the IEEE Engineering in Medicine and Biology Society (EMBC)*.

[12] Ruifrok AC, Johnston DA (2001). Quantification of histochemical staining by color deconvolution. *Anal. Quant. Cytol. Histol.*.

[13] Waugh DJJ, Wilson C (2008). The interleukin-8 pathway in cancer. *Clin. Cancer Res.*.

[14] Aalinkeel R, Nair MP, Sufrin G (2004). Gene expression of angiogenic factors correlates with metastatic potential of prostate cancer cells. *Cancer Res.*.

[15] Waugh DJ, Wilson C (2008). The interleukin-8 pathway in cancer. *Clin. Cancer Res.*.

[16] Seaton A, Scullin P, Maxwell PJ (2008). Interleukin-8 signaling promotes androgen-independent proliferation of prostate cancer cells via induction of androgen receptor expression and activation. *Carcinogenesis*.

[17] Chadha KC, Miller A, Nair BB, Schwartz SA, Trump DL, Underwood W (2014). New serum biomarkers for prostate cancer diagnosis. *Clin. Cancer Investig. J.*.

[18] Caruso DJ, Carmack AJ, Lokeshwar VB, Duncan RC, Soloway MS, Lokeshwar BL (2008). Osteopontin and interleukin-8 expression is independently associated with prostate cancer recurrence. *Clin. Cancer Res.*.

[19] Zabransky DJ, Smith HA, Thoburn CJ (2012). Lenalidomide modulates IL-8 and anti-prostate antibody levels in men with biochemically recurrent prostate cancer. *Prostate*.

